# Edema of the Scrotum and Penile Shaft: An Uncommon Initial Presentation of Acquired Angioedema With Low C1-Inhibitor

**DOI:** 10.1155/2024/9172329

**Published:** 2024-09-11

**Authors:** Meghan V. Matheny, Timothy Craig, Joseph Y. Clark

**Affiliations:** ^1^ School of Medicine Penn State University, 500 University Drive, Hershey, Pennsylvania 17033, USA; ^2^ Section of Allergy Asthma & Immunology Pennsylvania State University School of Medicine, 500 University Drive, Hershey, Pennsylvania 17033, USA; ^3^ Vinmec International Hospital, 458 P. Minh Khai, Khu đô thị Times City, Hai Bà Trưng, Hanoi 100000, Vietnam; ^4^ Department of Urology Pennsylvania State University School of Medicine, USA

**Keywords:** acquired angioedema, acquired angioedema with low C1-inhibitor, angioedema, penile angioedema, scrotum angioedema

## Abstract

Acquired angioedema with low C1-inhibitor (AAE-C1-INH) is a rare disorder characterized by an acquired deficiency in the C1 esterase inhibitor (C1-INH). This case report describes a 79-year-old patient presenting to the emergency department for painless swelling of his scrotum, penile shaft, and left lower and upper extremities with lab values consistent with acquired angioedema without identifiable lymphoreticular or rheumatic disorder on history, exam, or total body PET scan. Proper diagnosis of AAE-C1-INH is essential to prevent life-threatening airway compromise, ensure proper therapy, and exclude lymphoreticular disorders as the etiology of AAE-C1-INH.

## 1. Introduction

Acquired angioedema with low C1-inhibitor (AAE-C1-INH) is an extremely rare disorder characterized by an acquired deficiency of C1 esterase inhibitor (C1-INH), recurrent episodes of angioedema, and increased activation of the classical complement pathway and contact system [1]. Importantly, AAE-C1-INH must be differentiated from histamine-induced angioedema and hereditary forms of C1-INH deficiency (hereditary angioedema [HAE]). HAE typically presents with an earlier age of onset, family history, and mutations within the SERPING1 gene [2]. Deficiency of the C1-INH leads to inappropriate activation of the contact-kinin system, resulting in bradykinin-mediated blood vessel leakage associated with localized swelling referred to as angioedema [3]. Clinically, the angioedema is nonpitting, nonpruritic, and affects the skin, abdomen, upper airway, and genitals [4]. Early diagnosis of both HAE and AAE-C1-INH is essential, as swelling can impact the mucosa of the gastrointestinal and upper respiratory tract, leading to bowel obstruction or life-threatening airway compromise [5]. AAE-C1-INH specifically has been reported to have a higher prevalence of recurrence involving the face, tongue, and uvula [6]. The etiology of AAE-C1-INH is not fully understood, but it is commonly associated with an underlying lymphoreticular/rheumatic disorder. The presence of neutralizing autoantibodies against C1-INH has also been described in some patients with AAE-C1-INH [7]. Diagnosis of AAE-C1-INH is made through clinical suspicion and lab testing showing low C1-INH protein and function, C1q, and C4 levels. Treatment consists of therapy for attacks of angioedema (on-demand therapy [ODT]), long-term suppression of attacks (long-term prophylaxis [LTP]), and short-term prophylaxis (STP) to use to prevent swelling before a possible trigger such as surgery [8]. In this report, we describe a 79-year-old patient with his first episode of angioedema presenting with painless swelling of his scrotum and penile shaft with lab values consistent with acquired angioedema without identifiable lymphoreticular or rheumatic disorder.

## 2. Case Presentation

**Figure 1:**
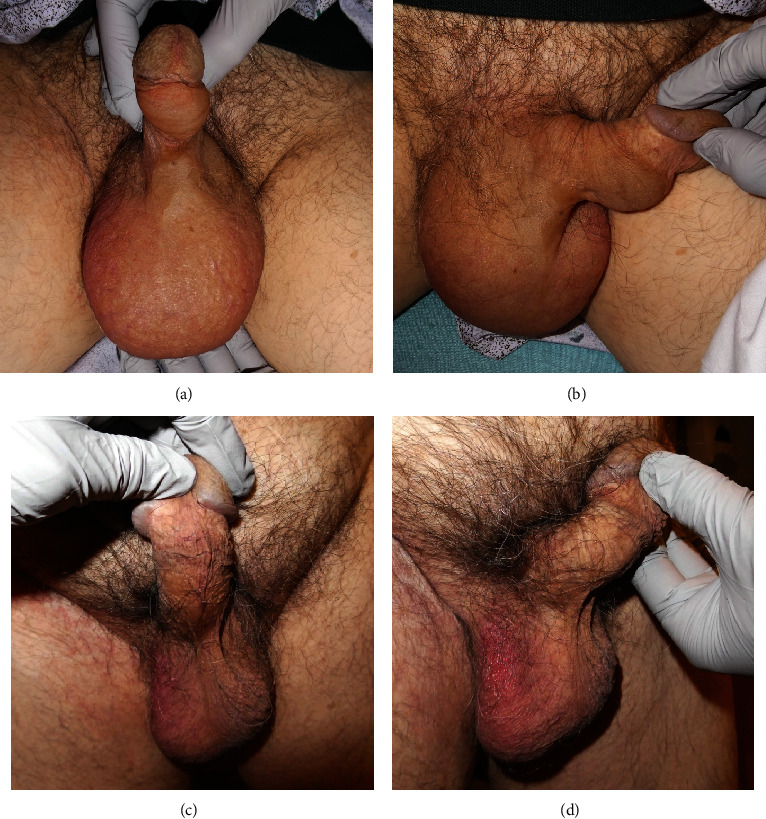
Acute onset scrotal and ventral penile nonpitting edema. (a) During the episode, front view. (b) During the episode, side view. (c) After the resolution of the episode, 1 week later, front view (d). After the resolution of the episode, 1 week later, side view.

**Figure 2:**
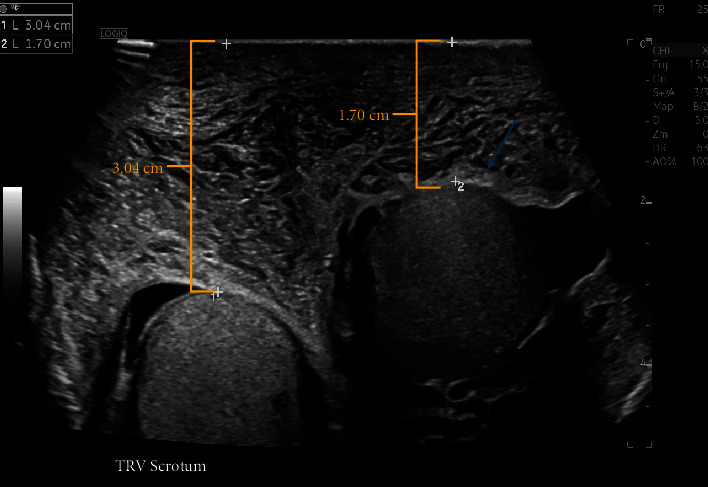
Scrotal ultrasound showing moderate to severe scrotal wall thickening consistent with diffuse scrotal edema.

The patient presented to the emergency department with painless bilateral scrotal and penile shaft swelling without warmth or erythema that had begun acutely that morning (Figures 1(a) and 1(b)). He was treated initially for potential allergic etiology with one dose of diphenhydramine (25 mg, PO) and methylprednisolone (125 mg, IV) which did not improve the swelling. Over the course of the subsequent hours, his left foot, ankle, and hand also became swollen. The patient reported no other associated symptoms such as nausea, vomiting, fever, trauma to the region, or insect bites, and the swelling did not interfere with voiding. Upon consultation with urology, scrotal ultrasound with Doppler was recommended, and it revealed only nonspecific findings of diffuse scrotal edema and hyperemia and bilateral small hydroceles (Figure 2). Lower extremity duplex ultrasound ruled out deep vein thrombosis as the cause of the swelling. The urology team concluded no acute urologic interventions were indicated at that time.

After excluding the need for surgery, allergy was consulted for assessment of the C1-esterase-mediated angioedema (Table 1). The results of this workup were consistent with edema due to deficiency in the C1-INH. Importantly, the administration of C1-INH protein led to a marked improvement in swelling. HAE was considered but deemed unlikely due to the age of the first presentation and lack of family history despite the normal level of C1q. Further genetic testing revealed no pathogenic genetic variants known to cause HAE in the commonly associated genes ANGPT1, FT2, PLG, and SERPING1. The patient additionally denied the use of any ACE inhibitors.

The patient was evaluated for underlying lymphoreticular disorder due to its strong association with acquired C1-INH deficiency. Serum protein electrophoresis and immunofixation electrophoresis showed no monoclonal immunoglobulins. An abdominal ultrasound from 1 month after the episode showed an unremarkable liver and spleen. The patient denied any B symptoms such as weight loss, fevers, night sweats, and bone or joint pain. The patient had no family history of rheumatologic disease and no increase in ESR or C-reactive protein. Full-body fluorine-18 fluorodeoxyglucose (18F-FDG) positron emission tomography (PET)/computed tomography (CT) showed no evidence of FDG-avid nodal or visceral disease (Table 2). On follow-up with urology, 1 week after the initial presentation and treatment with C1-INH, the swelling had completely resolved (Figures 1(c) and 1(d)). On a 2-week follow-up with allergy, the C1-INH level was still low, but the other lab values had stabilized on therapy (Table 1).

## 3. Discussion

Investigation of more common causes of scrotal swelling (torsion of the testis, hydrocele, varicocele, trauma, and tumor) with ultrasound was essential before consideration of a rare disorder such as AAE-C1-INH. The patient denied any known trauma to that region or pain-making torsion and trauma-unlikely etiologies. The ultrasound did show bilateral small hydroceles; however, the normal physiologic hydroceles were not felt to be related to the scrotal edema. Additionally, the progression of the edema to include the left leg and foot made a local pathology unlikely. Finally, the biochemical tests and the response to the C1-inhibitor protein suggested bradykinin as the mediator. One limitation of our investigation was that we did not obtain a D-dimer level due to the low index of suspicion for clotting etiology. Elevation in D-dimer is seen during acute attacks in both patients with HAE and AAE-C1-INH because the contact and fibrinolytic pathways are interconnected. The activated fibrinolytic pathway can lead to Activated Factor 12, leading to the generation of bradykinin.

Allergic angioedema is more common than any of the bradykinin-mediated varieties, but lack of histamine-related symptoms such as urticaria and lack of response to corticosteroids and antihistamine therapy distract from this diagnosis. Some medications, most commonly ACE inhibitors but also dipeptidyl peptidase-IV inhibitors and recombinant tissue plasminogen activators, have been associated with acute-onset bradykinin-related angioedema [9]. The patient was not on any of these medications, making this an unlikely etiology for his edema. Medication-associated angioedema would also not account for the patient's low C1-inhibitor level.

Diagnosis of AAE-C1-INH requires exclusion of HAE through differences in clinical presentation, older onset, lack of family history, and abnormal C1q in most cases of AAE-C1-INH. Rarely, when the case or the biochemical tests are not diagnostic, investigation for classic genetic mutations associated with HAE may be necessary. Importantly, this patient had no prior episodes of angioedema before the age of 79, no family history of angioedema, and failed to respond to antihistamines and corticosteroids. These first two factors make HAE, which typically presents in the first two decades of life in patients with a positive family history of angioedema, much less likely [10]. Additionally, this patient underwent thorough genetic testing for all known HAE-related genes which did not show evidence of any mutation. A complete rule out of HAE is not possible without full genome sequencing to evaluate for disease-associated mutations within introns. However, the other patient factors do not support the need for further evaluation. Therefore, the label of acquired angioedema with low C1-INH provides the best explanation for this patient's presentation.

Despite this diagnosis, the trend toward normalization of his C1-inhibitor protein and C4 is very unusual for AAE-C1-INH with low C1-inhibitor. We initially hypothesized this trend was due to the use of corticosteroids causing decreased autoantibodies allowing recovery of his biochemical tests and the administration of C1-inhibitor treatment which increased C1-inhibitor levels. However, only one dose of corticosteroids was administered on admission, making this an unlikely explanation for the recovery of the laboratory values. Therefore, we are unsure of the etiology of this trend. Finally, his C1q being normal in AAE-C1-INH with low C1-inhibitor is not unusual. Although 70% have a low C1q, 30% fail to demonstrate this marker [6].

The absence of underlying lymphoreticular or rheumatologic disorder discovered in this patient may seem like a major limitation to the conclusive diagnosis of acquired angioedema. However, it is not unusual for the AAE-C1-INH to present before lymphoma or apparent autoimmune disease [3]. Monitoring of the patient in this case report over the next several years may reveal a new lymphoreticular disorder not shown on initial testing.

Current treatment of AAE-C1-INH is based on guidelines for HAE due to similar disease mechanisms and clinical presentation [11]. Because early intervention during acute attacks is associated with better symptom resolution, on-demand self-administrable therapies are essential in the prevention of life-threatening facial and laryngeal edema [12, 13]. The first-line agents for acute episodes decrease bradykinin production or function and include intravenous C1-INH, ecallantide, and icatibant [11]. Treatment with exogenous C1-INH directly increases plasma concentration, successfully decreasing bradykinin through all C1-INH-related pathways [14]. Inhibition of kallikrein by ecallantide reduces the cleavage of kininogen to bradykinin. Icatibant is a competitive antagonist for the Bradykinin B2 receptor, decreasing vascular permeability and vasodilation. If these first-line agents are not available, solvent detergent–treated plasma (SDP) and fresh frozen plasma can also be used [11].

Certain circumstances that may result in damage to the upper airway such as mechanical intubation, scope, or surgical or dental trauma may necessitate additional STP for patients with AAE-C1-INH. Preprocedural prophylaxis has been shown to reduce the risk of angioedema near the site of intervention [15]. In anticipation of an angioedema-associated surgical or dental procedure, intravenous plasma-derived C1-INH is recommended as first-line STP. Attenuated androgens such as danazol are an accepted alternative for STP but require starting therapy 5 days before the procedure [16].

Ideally, physicians should strive to reduce the number of acute attacks in patients with AAE-C1-INH using LTP. Therapies include intravenous C1-INH, or subcutaneously C1-INH twice weekly [10]; lanadelumab, a subcutaneously injectable antiactive plasma kallikrein monoclonal antibody; and berotralstat, an oral plasma kallikrein inhibitor [17, 18]. Unfortunately, some patients with AAE-C1-INH associated with lymphoma become resistant to plasma-derived C1-INH due to the high rate of catabolism of C1-INH protein [6]. More recent treatment directed at the underlying lymphoma with rituximab has shown to provide effective long-term prevention of angioedema attacks and is the preferred therapy at this time [19].

## 4. Conclusion

This patient is important for reporting because the rarity of AAE-C1-INH means many healthcare professionals have limited knowledge regarding its diagnosis and treatment. Proper diagnosis of AAE-C1-INH will allow for early appropriate treatment, decreasing the risk of life-threatening airway compromise and skin and abdominal symptoms and avoiding unnecessary use of corticosteroid and antihistamine therapies. The diagnostic pathway followed for this patient could provide a framework for the investigation of atypical genital swelling that is not well explained by more common etiologies. Additionally, for some patients, AAE-C1-INH may be the first clinical manifestation of their underlying lymphoreticular disorder. Proper workup, knowledge of angioedema, and a high index of suspicion would result in efficient care of patients with this condition.

## Figures and Tables

**Table 1:** Patient lab values for angioedema diagnosis.

	**C1-INH** **Protein level (reference range: 21–38 mg/dL)**	**C1-INH** **Functional level (reference range: >67%)**	**C4** **Protein level (reference range: 10–40 mg/dL)**	**C1q** **Protein level (reference range: 109–242 *μ*g/mL)**
Emergency department	14 (low)	41 (low)	<3 (low)	108 (borderline)
2-week follow-up	19 (low)	69 (borderline)	17 (normal)	147 (normal)

**Table 2:** Imaging and laboratory evaluation for the underlying lymphoreticular disorder.

**Test**	**Purpose**	**Result**
Serum protein	Evaluation for multiple myeloma	6.8 g/dL
Albumin		4.3 g/dL
M-spike, quantitative/creatine		Urine protein concentration too low for accurate quantitation (milligrams per deciliter)
M-spike, quantitative		
Urine immunofixation		No monoclonal immunoglobulins detected
Serum protein electrophoresis		No monoclonal immunoglobulins detected
C-reactive protein	Evaluation for inflammatory process or rheumatologic disorder	<0.3 mg/dL
Erythrocyte sedimentation rate		12 mm/h
Abdominal ultrasound	Evaluation for lymphoma	Normal abdominal ultrasound
PET CT whole body		No FDG avid nodal or visceral disease is identified

## Data Availability

The data that support the findings of this study are available from the corresponding author, J.Y.C., upon reasonable request.

## Nomenclature

AAE-C1-INH: acquired angioedema with low C1-inhibitor
C1-INH: C1 esterase inhibitor
HAE: hereditary angioedema
LTP: long-term prophylaxis
ODT: on-demand therapy
STP: short-term prophylaxis
